# Schwann cells promote post-traumatic nerve inflammation and neuropathic pain through MHC class II

**DOI:** 10.1038/s41598-017-12744-2

**Published:** 2017-10-02

**Authors:** Maike Hartlehnert, Angelika Derksen, Tim Hagenacker, David Kindermann, Maria Schäfers, Mathias Pawlak, Bernd C. Kieseier, Gerd Meyer zu Horste

**Affiliations:** 1 Department of Neurology, University Hospital Münster, Münster, Germany; 2 Department of Neurology, Heinrich-Heine-University, Medical Faculty, Düsseldorf, Germany; 3 Department of Neurology, University of Duisburg-Essen, Essen, Germany; 4 Evergrande Center for Immunologic Diseases, Harvard Medical School and Brigham and Women’s Hospital, Boston, MA USA

## Abstract

The activation of T helper cells requires antigens to be exposed on the surface of antigen presenting cells (APCs) via MHC class II (MHC-II) molecules. Expression of MHC-II is generally limited to professional APCs, but other cell types can express MHC-II under inflammatory conditions. However, the importance of these conditional APCs is unknown. We and others have previously shown that Schwann cells are potentially conditional APCs, but the functional relevance of MHC-II expression by Schwann cells has not been studied *in vivo*. Here, we conditionally deleted the MHC-II β-chain from myelinating Schwann cells in mice and investigated how this influenced post-traumatic intraneural inflammation and neuropathic pain using the chronic constriction injury (CCI) model. We demonstrate that deletion of MHC-II in myelinating Schwann cells reduces thermal hyperalgesia and, to a lesser extent, also diminishes mechanical allodynia in CCI in female mice. This was accompanied by a reduction of intraneural CD4+ T cells and greater preservation of preferentially large-caliber axons. Activation of T helper cells by MHC-II on Schwann cells thus promotes post-traumatic axonal loss and neuropathic pain. Hence, we provide experimental evidence that Schwann cells gain antigen-presenting function *in vivo* and modulate local immune responses and diseases in the peripheral nerves.

## Introduction

A central element of all adaptive immune responses is recognition of antigens by T cells. CD4+ T helper cells recognize their cognate antigen via class II major histocompatibility complexes (MHC-II) presented on the surface of antigen presenting cells (APCs). While MHC class I is expressed by all nucleated cells, MHC-II is generally only expressed by professional APCs (such as dendritic cells, macrophages or B cells)^1,2^. However, under inflammatory conditions, other cell types such as vascular endothelial cells and muscle cells can upregulate MHC-II expression and subsequently activate CD4+ T cells (under defined culture conditions *in vitro* and after injection of exogenous antigen *in vivo*)^3–6^. Such cell types have been described as conditional APCs and could potentially modulate local inflammatory diseases^7^, but strong *in vivo* evidence for a functional relevance of MHC-II expression by conditional APCs is lacking.

We and others have previously shown that the myelin-forming glial cells of the peripheral nervous system (PNS) - named Schwann cells - can also gain MHC-II expression after traumatic^8^ and inflammatory injury^9^ and may present antigens *in vitro* ^10,11^. These surprising findings suggest a previously unappreciated function of Schwann cells as conditional APCs^9^ in addition to their function in myelination and axonal support^12^. However, like other conditional APCs, the functional relevance of MHC-II expression by Schwann cells has never been confirmed *in vivo* and could constitute either an important modulator of disease or an irrelevant epiphenomenon of inflammation. Here we tested this ‘conditional APC theory’ by using the PNS as a model system to delete APC function in a rigorous and genetically defined manner *in vivo*.

Previous data indicate that Schwann cells upregulate MHC-II under conditions inducing post-traumatic neuropathic pain in female rats^8^. Neuropathic pain is a debilitating condition associated with pain without adequate stimulus and due to decreased nociceptive thresholds^13^. Such triggering of a pain response from stimuli, which do not normally provoke pain, is termed allodynia and is a frequent clinical sign of neuropathic pain^14^. Neuropathic pain can result from various types of nerve injury, is common and affects up to one in twenty people worldwide – with a higher prevalence among women compared to men^15,16^. Evidence suggests that dysfunction of neurons and glial cells, but also immune cells and cytokines, participate in the development and chronicity of neuropathic pain^17^. In our study, we used the reliable and well-established chronic constriction injury (CCI) model of the sciatic nerve to test the functional relevance of MHC-II expression in Schwann cells in the context of neuropathic pain *in vivo*.

By conditionally deleting the MHC-II β-chain specifically in myelinating Schwann cells, we demonstrate that MHC-II expression by myelinating Schwann cells promotes post-traumatic CD4+ T cell infiltration and axonal degeneration and increases thermal hyperalgesia and mechanical allodynia in female mice *in vivo*. The ability of Schwann cells to present antigens thus promotes post-traumatic neuropathic pain. With these observations, we provide strong experimental evidence that Schwann cells can function as conditional APCs and - in a more general context - our data support the conditional APC paradigm in a living organism.

## Results

### A novel tool to conditionally delete MHC-II in Schwann cells *in vivo*

**Figure 1:**
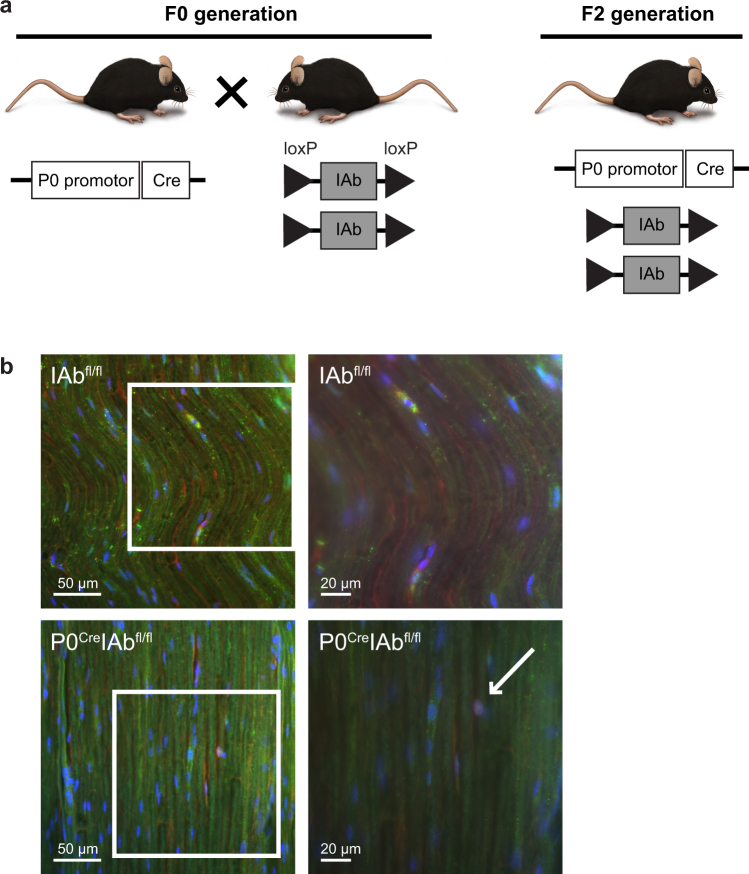
Characterization of the P0^Cre^IAb^fl/fl^ mouse line. (**a**) P0^Cre^ mice were crossed with IAb^fl/fl^ mice to generate homozygous P0^Cre^IAb^fl/fl^ mice. Wildtype IAb^fl/fl^ mice served as controls in all experiments. Acknowledgements for drawings of mice to Prof. Dr. Sven Meuth and Heike Blum, Institute for Translational Neurology, University Hospital Münster, Münster, Germany. (**b**) One week after chronic constriction injury, longitudinal paraffin sections (thickness 6 µm) of the sciatic nerve from wildtype IAb^fl/fl^ mice (top panels) and P0^Cre^IAb^fl/fl^ mice (bottom panels) were stained against S100 (green signal), MHC class II (MHC-II; red signal) using fluorescently labelled secondary antibodies; nuclei were stained with DAPI. White arrow indicates an MHC-II expressing macrophage. Right panels depict higher magnifications of the areas indicated in the left panels.

Myelin-forming Schwann cells can gain MHC-II expression after traumatic^8^ and inflammatory injury^9^, but the functional relevance of this expression *in vivo* has remained questionable. We therefore conditionally deleted the MHC-II β-chain in myelinating Schwann cells by crossing P0^Cre^ mice, which express the Cre recombinase in myelinating Schwann cells^18^, with IAb^fl/fl^ mice carrying a loxP site flanking exon 1 of the *IAb* gene (gene symbol *H2-Ab1*), which encodes the MHC-II β-chain in this mouse strain^19^ (Fig. 1a). To confirm the selective loss of MHC-II in Schwann cells of homozygous P0^Cre^IAb^fl/fl^ mice and thereby to confirm the functionality of our novel P0^Cre^IAb^fl/fl^ mouse line, we performed immunohistochemistry of peripheral nerves before and after chronic constriction injury (CCI) to the peripheral nerve. CCI is known to induce MHC-II expression in Schwann cells^8^. Before CCI, we did not detect MHC-II expressing myelinating Schwann cells (identified by S100 staining) in either IAb^fl/fl^ or P0^Cre^IAb^fl/fl^ mice (Supplementary Figure S1). After CCI, Schwann cells did express MHC-II in IAb^fl/fl^ control mice, but they did not express detectable MHC-II in P0^Cre^IAb^fl/fl^ mice (Fig. 1b). Although devoid of MHC-II expression in Schwann cells, the P0^Cre^IAb^fl/fl^ mice did express MHC-II in macrophages (white arrow in Fig. 1b) located in the injured peripheral nerve. This shows that deleting MHC-II was successful in myelinating Schwann cells and was specific to this cell type. This argues for the adequacy of our genetic approach.

### Schwann cell–derived MHC-II promotes post-traumatic axonal loss in female mice

We next used this novel tool to test the hypothesis that MHC-II expressed by Schwann cells modulates post-traumatic axonal injury by influencing local immune reactions in the peripheral nerve *in vivo*. As previous studies had preferentially used female animals to study adaptive immunity in traumatic nerve injury^8,20^, we also utilized female mice, on which we performed CCI of the sciatic nerve (Supplementary Figure S2)^8^. This CCI model causes both axonal degeneration and post-traumatic neuropathic pain within 7 days (Supplementary Figure S3) and thus allows the study of both phenomena in a tightly controlled fashion^21^. We performed CCI in female control IAb^fl/fl^ and MHC-II deficient P0^Cre^IAb^fl/fl^ mice and, after 7 days, analyzed axonal loss by neurofilament staining and myelin maintenance.

**Figure 2:**
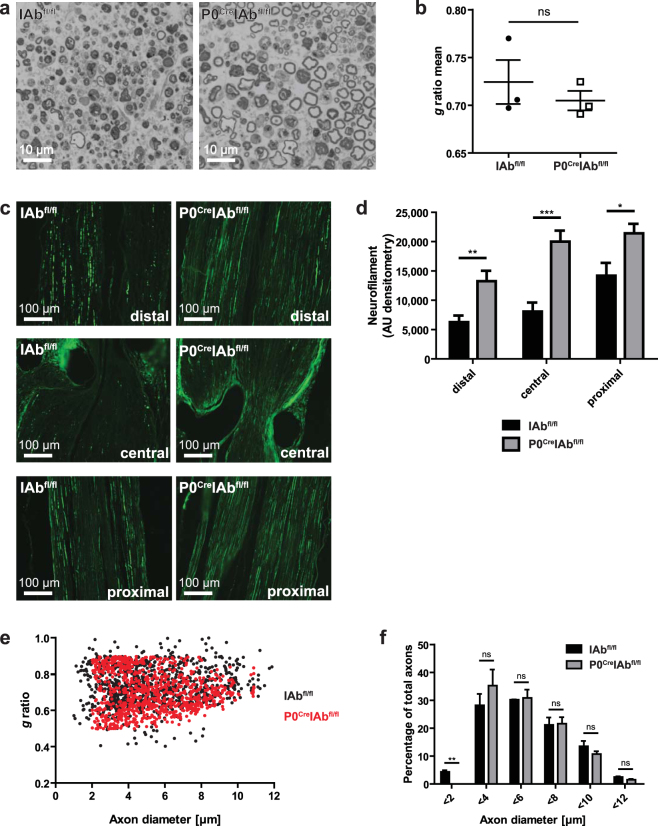
Schwann cell–specific MHC class II deficiency reduces post-traumatic axonal loss in female mice. (**a**) Sciatic nerves from wildtype IAb^fl/fl^ female mice (left panel) and P0^Cre^IAb^fl/fl^ female mice (right panel) were dissected 7 days after chronic constriction injury (CCI) of this nerve for histological examination. Semi-thin sections cut 2 mm distal to the crush site were Toluidine-Blue stained. (**b**) Axon circumference and myelin circumference were manually measured for >100 axons per nerve section using ImageJ. The *g* ratio was calculated by dividing axon circumference by myelin circumference and average *g* ratio values were calculated for each mouse. The term ns means not significant. (**c**) Paraffin sections (6 µm) of longitudinally embedded sciatic nerves were stained with anti-neurofilament light chain and secondary fluorescent antibodies at sites distal, central and proximal to the CCI lesion. One representative of five female animals is depicted in *a* and *c*. (**d**) Neurofilament staining intensity described in *c* was quantified in arbitrary units of densitometry (AU) (n = 5 female animals per group). *p-value < 0.05, **p-value < 0.01, ***p-value < 0.001. (**e**) The *g* ratios of each individual axon (>100 axons per mouse) was plotted against its corresponding axon diameter in IAb^fl/fl^ (black dots) and P0^Cre^IAb^fl/fl^ (red dots) female mice. (**f**) Axons were grouped based on their diameter and the percentage of axons per size group was plotted using the data depicted in *b* and *e* from IAb^fl/fl^ and P0^Cre^IAb^fl/fl^ female mice (n = 3 per group). **p-value < 0.01, ns not significant.

We found that Schwann cell–restricted MHC-II deficiency in female P0^Cre^IAb^fl/fl^ mice did not alter the proportion of myelinated axons (Fig. 2a) or alter the myelin thickness quantified by *g* ratio measurements (axonal diameter divided by myelin diameter) distal to the site of injury (Fig. 2b; *p*-value = 0.4809). We then performed neurofilament staining and found that this surrogate marker of axonal integrity and number of axons^22,23^ was present at higher densities in P0^Cre^IAb^fl/fl^ mice than in IAb^fl/fl^ mice (Fig. 2c,d). This was apparent at locations distal (*p*-value = 0.0022), central (*p*-value < 0.0001) and proximal (*p*-value = 0.0116) to the injury site (Fig. 2d). Together, these results indicate that MHC-II in Schwann cells promotes post-traumatic axonal loss but does not affect myelin loss. We next plotted axonal diameters against the *g* ratio and found no apparent difference in the constant ratio between axonal diameter and myelin sheath thickness between genotypes (Fig. 2e). The overall size distribution of axons was also not different between genotypes (Fig. 2f). However, we found a significantly lower proportion of small-caliber axons (axon diameter < 2 µm) in P0^Cre^IAb^fl/fl^ mice (*p*-value = 0.0018) than in control mice. This suggests that Schwann cell–specific MHC-II does not influence the loss of myelin, but does promote post-traumatic axonal loss; it also suggests that larger axons may be preferentially affected in this injury-driven model.

### Schwann cell–derived MHC-II promotes nerve inflammation, post-traumatic thermal hyperalgesia and mechanical allodynia in female mice

**Figure 3:**
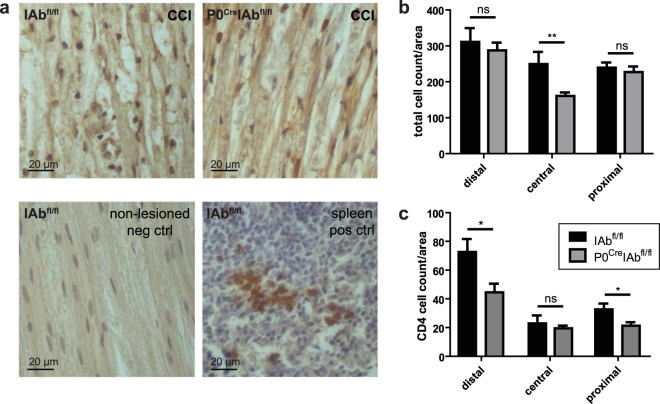
Reduced post-traumatic CD4+ cells in the absence of Schwann cell–specific MHC class II in female mice. (**a**) Seven days after chronic constriction injury (CCI), longitudinal paraffin embedded sciatic nerve sections (6 µm) from wildtype IAb^fl/fl^ female mice (left top panel) and P0^Cre^IAb^fl/fl^ female mice (right top panel) were cut at distal, central and proximal sites relative to the CCI lesion; sections were stained against CD4 using DAB, and nuclei were stained with Hemalaun. A representative distal section of one of the five animals tested is depicted. Sections of an uninjured “non-lesioned” nerve (left bottom panel) and of a healthy spleen (right bottom panel) from wildtype IAb^fl/fl^ mice stained analogous to the CCI sections serve as negative and positive control of the α-CD4 DAB-staining, respectively. (**b**,**c**) The number of total cells (**b**) and of total CD4+ cells (**c**) per section was manually counted using ImageJ. Average values of 5 female animals per group are depicted. *p-value < 0.05, **p-value < 0.01, ns not significant.

The function of MHC-II is to present antigens to CD4+ T helper cells. We therefore tested how MHC-II expression in Schwann cells affected the local presence of CD4+ T helper cells after traumatic nerve injury. Taking an immunohistochemical approach, we found a decreased staining intensity for CD4 in the peripheral nerves of female P0^Cre^IAb^fl/fl^ mice compared to IAb^fl/fl^ mice (Fig. 3a). We next quantified this observation by manually counting intraneural cells. We found that both the total number of intraneural cells (Fig. 3b) and the number of CD4+ T cells (Fig. 3c) was lower in P0^Cre^IAb^fl/fl^ mice than in IAb^fl/fl^ mice. Additionally, there were fewer intraneural cells at the site of injury in P0^Cre^IAb^fl/fl^ mice (Fig. 3b central; *p*-value = 0.039), while the number of CD4+ T cells was lower in proximal (*p*-value = 0.0154) and distal (*p*-value = 0.0128) areas (Fig. 3c) of P0^Cre^IAb^fl/fl^ mice compared to in controls. There was no difference in the number of CD4+ T cells in the center of the lesion, but this could have been due to the destruction of tissue at the site of injury. Overall, this suggests that Schwann cell–derived MHC-II locally promotes the infiltration or proliferation of CD4+ T cells in the peripheral nerve in female mice.

**Figure 4:**
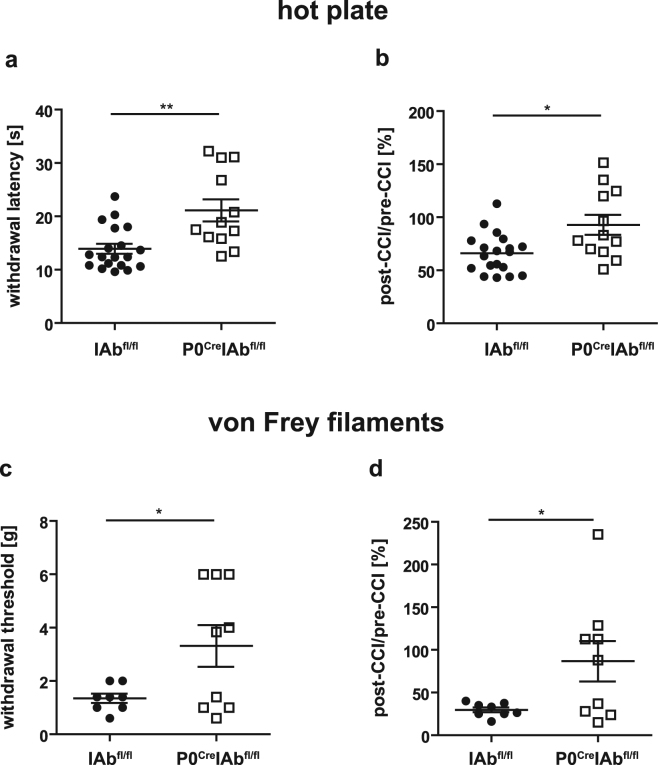
Schwann cell–specific MHC class II deficiency reduces post-traumatic thermal hyperalgesia and mechanical allodynia in female mice. (**a**) Thermal hyperalgesia was tested 7 days after chronic constriction injury (CCI) by placing the mouse’s hind paw from the lesioned side onto a hot plate (temperature 40 °C) and recording the latency (in seconds) until paw withdrawal (see methods). The latency, averaged between three technical repeats, is depicted and every symbol indicates one mouse. Note that *increased* latency reflects *decreased* hyperalgesia. (**b**) The post-CCI latency was divided by the average pre-CCI latency in every mouse and is expressed as a proportion of pre-CCI latency. (**c**) Mechanical allodynia was measured with von Frey filaments 7 days after CCI by pressing von Frey filaments of increasing strength against the plantar surface of the hind paw on the lesioned side and recording the thickness of filaments that induced paw withdrawal reaction. The corresponding force (in grams) was calculated (see methods). Note that *increased* threshold reflects *decreased* allodynia. (**d**) The post-CCI force was divided by the average pre-CCI force and is expressed as a proportion of pre-CCI force. Data were compared using unpaired *t*-test with Welch’s correction. *p-value < 0.05, **p-value < 0.01. Pooled data from three (hot plate) and two (von Frey filaments) independent experiments, respectively, with in total ≥8 female mice per group (wildtype IAb^fl/fl^ vs. P0^Cre^IAb^fl/fl^ mice), are depicted.

Post-traumatic neuropathic pain is enhanced by local inflammation^24^, and a previous study found that neuropathic pain depends on adaptive immunity in female mice^20^. We initially screened mice of both sexes, and male mice did not show any difference in mechanical allodynia and thermal hyperalgesia between genotypes in preliminary experiments (data not shown). We therefore focused our analysis on female mice: We analyzed how the reduced T cell content and enhanced axonal maintenance in P0^Cre^IAb^fl/fl^ mice influenced post-traumatic neuropathic pain in female mice. We used hot plate and von Frey filament testing to quantify thermal hyperalgesia and mechanical allodynia, respectively (Supplementary Figure S3). We found that the threshold for paw withdrawal to heat was significantly higher in female P0^Cre^IAb^fl/fl^ mice than in female IAb^fl/fl^ mice using both absolute (Fig. 4a, *p*-value = 0.0065) and relative quantification (Fig. 4b, *p*-value = 0.0202), indicating that deleting MHC-II from peripheral myelination Schwann cells led to a decreased sensitivity to noxious thermal stimuli and decreased thermal hyperalgesia. This was apparent in each independent experiment and in a summarized analysis (depicted in Fig. 4a and b). Withdrawal thresholds to von Frey filaments were also increased in P0^Cre^IAb^fl/fl^ mice compared to IAb^fl/fl^ mice when examining absolute values (Fig. 4c, *p*-value = 0.0398) and using relative quantification (Fig. 4d, *p*-value = 0.0436) in a pooled analysis. This trend did not reach our significance threshold, indicating that the sensitivity to mechanical stimuli and mechanical allodynia are less affected by loss of MHC-II on Schwann cells. In sum, our data indicate that Schwann cell–derived MHC-II promotes post-traumatic thermal hyperalgesia and, to a lesser extent, mechanical allodynia, thus promoting post-traumatic neuropathic pain.

## Discussion

Overall, we demonstrate that MHC-II expression by Schwann cells promotes post-traumatic inflammation in the peripheral nerve and subsequently exacerbates axonal loss and neuropathic pain in female mice. Our study has several important implications. First, it confirms earlier descriptive and cell culture–based studies that MHC-II expressed by Schwann cells confers pro-inflammatory functions^10,11^. Second, it suggests that blocking MHC-II could constitute a potential therapeutic approach if it is done early during the development of post-traumatic neuropathic pain. Potential pharmaceutical candidates might be compounds that redistribute MHC-II from the cell surface^25^. Third, to the best of our knowledge, this study provides the first rigorous *in vivo* evidence that MHC-II expression by non-professional APCs does participate in disease manifestation. On a broader scale, our study supports the paradigm that some cell types can become conditional APCs under disease conditions and participate in the local manifestation of disease within a tissue. It is intriguing to speculate whether genetically targeting MHC-II deficiency to other cell types that may serve as conditional APCs (e.g. muscle cells or endothelial cells) could modulate organ-specific inflammatory diseases due to trauma or autoimmunity.

Our study features a unique technical advantage over previous studies in that we used genetically defined conditional deletion of MHC-II in a cell type–specific manner. We inter-crossed the P0^Cre^ and IAb^fl/fl^ mouse lines and thereby restricted deletion of the MHC-II β-chain (encoded by the *IAb* gene; gene symbol *H2-Ab1*) to myelinating Schwann cells (targeted by the myelin protein zero (*Mpz* or P0) promoter). This approach leaves MHC-II expression intact on all professional APCs, which we confirmed by demonstrating an MHC-II expressing mononuclear cell in the P0^Cre^IAb^fl/fl^ mouse line (Fig. 1b). Other researchers previously crossed the IAb^fl/fl^ mouse line to other Cre driver lines and thereby successfully ablated functional MHC-II expression in diverse cell types such as innate lymphoid cells and B cells^26,27^. This supports that the IAb^fl/fl^ allele is indeed functional and can be used to delete MHC-II in a cell type–specific manner. In our experimental setting, we were able to specifically direct deletion in Schwann cells by using P0^Cre^, as this mouse line is an established tool to specifically target myelinating Schwann cells^28–30^. Thus, our mouse lines are well characterized and achieve successful deletion of MHC-II as intended. In accordance, we confirmed the selective loss of MHC-II on Schwann cells (Fig. 1) supporting the efficiency and specificity of our approach.

Schwann cells can be either myelinating or non-myelinating^12,31^, and the P0^Cre^ mouse line only targets myelinating Schwann cells because P0 expression is initiated only after myelination commences^32^. Interestingly, after CCI in mice without MHC-II on myelinating Schwann cells, unmyelinated small-caliber axons with a diameter <2 µm were significantly under-represented (Fig. 2f). The lack of small-caliber axons in P0^Cre^IAb^fl/fl^ mice can be interpreted to indicate either that 1) small axons are preferentially lost in P0^Cre^IAb^fl/fl^ mice, or that 2) large axons are preferentially maintained thereby shifting the relative distribution to larger sizes. If the first interpretation is correct, small-caliber axons could be more sensitive to local accumulation of CD4+ T cells promoted by MHC-II on myelinating Schwann cells and thus be preferentially lost in P0^Cre^IAb^fl/fl^ mice. This would indicate that CD4+ T cells activated by MHC-II on myelinating Schwann cells exert a negative bystander effect on non-myelinating Schwann cells: We speculate that the change in CD4+ T cell numbers (Fig. 3c) due to the lack of MHC-II expression in myelinating Schwann cells may modify the functionality of non-myelinating Schwann cells, which would subsequently and indirectly affect thermal hyperalgesia. Such an indirect effect is also consistent with the fact that thermal hyperalgesia is altered in the P0^Cre^IAb^fl/fl^ mouse line (Fig. 4a and b) but is mainly transduced by unmyelinated C-fibers that are not genetically targeted by the P0^Cre^ allele. We thus consider the first potential explanation to be more likely.

We found that there were fewer CD4+ T cells in P0^Cre^IAb^fl/fl^ mice compared to in control IAb^fl/fl^ animals (Fig. 3c; proximal and distal areas relative to CCI lesion), which is interesting because this cell type is activated by MHC-II. Also, we found that the total number of intraneural cells was unchanged (Fig. 3b). We speculate that the Schwann cell–specific deletion of MHC-II changes the activation of CD4+ T cells and secondarily alters the infiltration of other mononuclear cells.

We also found a decrease of total cell number (Fig. 3b), albeit not of CD4+ cell numbers (Fig. 3c), in the central part of the nerve (in P0^Cre^IAb^fl/fl^ mice). Morphology of this central part is directly impacted by CCI lesion, so this finding may represent a technical issue rather than biological phenomenon.

In this study, we used the traumatic CCI model to test the importance of antigen presentation as a core element of the adaptive immune system. We used neuropathic pain rather than a primarily immune driven disease in this mouse line for two reasons. First, although several different protocols to induce experimental autoimmune neuritis (EAN) in C57BL/6 mice have been published^33,34^, we found that none of these protocols were successful despite rigorous testing of several different antigenic peptides and adjuvants (unpublished observations). Second, a previous study had elegantly shown that Schwann cells strongly upregulate MHC-II after traumatic injury^8^. The CCI model of neuropathic pain thus provides a reliable model to address antigen presentation of Schwann cells *in vivo*. We focused our analysis on female mice based on previous findings that adaptive immunity modulates mechanical allodynia in female mice but not in male mice^20^. Furthermore, MHC-II expression in Schwann cells after experimental induction of post-traumatic neuropathic pain was previously observed in female rats^8^. In addition, our preliminary experiments indicated that Schwann cell–restricted loss of MHC-II does not affect neuropathic pain in male mice. This female-specific effect indicates that MHC-II dependent adaptive immunity promotes post-traumatic pain in a sex-specific way. Future studies would be required to elucidate how sex influences the interaction between adaptive immunity and the nervous system in neuropathic pain.

We chose the CCI model of neuropathic pain because it features a reproducible and defined onset and short duration. In fact, this model showed low inter- and intra-individual variability of measures of thermal hyperalgesia. Greater variability was seen when testing for mechanical allodynia, and statistically significant differences were only observed when merging experiments. Technical difficulties may account for this difference: Six von Frey filaments with increasing rigidity (see methods section) were used. Thus, the read-out is not a continuous parameter, and von Frey tests are more prone to bias than hot plate experiments as they require a dichotomous yes/no decision by the observer^35,36^. The higher inter-and intra-test variability of von Frey filament tests also explains why differences in mechanical allodynia reached our threshold of significance only in pooled data analysis.

We used the intensity of neurofilament staining as a surrogate marker of axonal integrity and axonal numbers. We did not count the total number of axons in semi-thin sections. Although our technical approach has limitations, it has been previously described and utilized to quantify intact axons in experimental paradigms similar to ours^22,23^.

Neuropathic pain is a common and severely disabling epiphenomenon of various types of injuries to the PNS. Schwann cells are known to *directly* control the development of neuropathic pain. For example, deficiency of components of the Neuregulin/ErbB signaling system, which determines myelination fate decisions of Schwann cells and myelin thickness^37–39^, results in enhanced mechanical allodynia^40^. In addition, researchers found that non-myelinating Schwann cells controlled thermal allodynia in a rodent model of HIV-associated neuropathy induced by treating mice expressing the viral protein gp120 in glial cells with an antiretroviral drug^41^. Here, we introduce an additional *indirect* mechanism of how Schwann cells instruct the development of neuropathic pain by altering the recruitment or local proliferation of T cells in the PNS. In addition, our findings lend support to the notion that anti-inflammatory drugs could reduce neuropathic pain in conditions that are not primarily immune mediated. Thus, future experiments should test whether drugs targeting adaptive immunity (e.g. tacrolimus) or treatments targeting the interaction between T cells and APCs can alleviate post-traumatic neuropathic pain.

Taken together, our findings suggest that Schwann cells control the development of neuropathic pain through a variety of mechanisms that may be independently exploitable for treatments.

## Materials and Methods

### Mice

P0^Cre^ mice^18^ were crossed with IAb^flox^ mice^19^ to generate mice heterozygous for the P0^Cre^ allele and homozygous for the IAb^flox^ allele; these mice are referred to as P0^Cre^IAb^fl/fl^ mice throughout the study. Littermate IAb^fl/fl^ mice negative for the P0^Cre^ allele served as controls in all experiments. Four independent experiments were performed. One experiment was performed with male mice and three independent experiments were performed with female mice. All depicted data are derived from experiments with female mice. Mice were used at 3–6 months of age. All animal experiments were approved by the responsible state authorities (LANUV NRW) and were performed in accordance with local guidelines and regulations.

### Chronic constriction injury (CCI)

For CCI, mice received intraperitoneal injections of Ketamin (5 mg/kg) and Xylazin (100 mg/kg); during narcosis, mice were kept on a 37 °C heated pad. CCI was performed as previously described^21,42^. Briefly, the fur was shaved off in the dorsal pelvic area. Then, after thorough alcoholic disinfection, an incision was made from the sciatic notch approximately 2–4 mm to the mid-thigh using scissors. The left sciatic nerve was exposed by partly removing the gluteal muscle from the sacral bone and bluntly separating the muscles below. Then, three consecutive ligatures (6–0 chromic gut suture) were tied loosely around the sciatic nerve with 2–3 mm distance between ligatures. Subsequently, lesions were closed by suturing in layers as required. The right sciatic nerve was exposed in every mouse as described above, but not injured, and served as a sham control.

### Hot plate experiments for thermal hyperalgesia testing

Hot plate experiments were performed as previously described^43^ with minor modifications. Briefly, a small metal hot plate with a diameter of 1 cm (“7341 Plantar Test”; Ugo Basile) was heated to a constant temperature of 40 °C and was then lightly pressed onto the plantar surface of the hind paw of each experimental mouse. The time until paw withdrawal was recorded and defined as the thermal threshold (unit: seconds) and the experiment was repeated with the other hind paw. Both hind paws were measured three times per session with a recovery time of five minutes between measurements. Mean values of the technical replicates per session were calculated. All testing was performed by the same blinded investigator.

### Von Frey filaments for mechanical allodynia testing

Mechanical allodynia was tested according to a previously described method^44^ with minor modifications. Briefly, mice were placed on a metal mesh and von Frey filaments (Ugo Basile) were pushed against the plantar surface of the hind paw for one second. Six different filaments with increasing force were used. The filament that triggered the mouse to move its hind paw was defined as the mechanical threshold. The categories and corresponding weights of the von Frey filaments were as follows: 3.61 von Frey ≈ 0.4 g; 3.84 von Frey ≈ 0.6 g; 4.08 von Frey ≈ 1 g; 4.17 von Frey ≈ 1.4 g; 4.31 von Frey ≈ 2 g; 4.56 von Frey ≈ 4 g. If the 4.56 von Frey filament did not cause any movement, a 4.74 von Frey (≈6 g) was defined as the mechanical threshold (unit: grams). Both hind paws were measured three times per session with a recovery time of one minute between measurements. All tests were performed by the same blinded investigator.

### Pain sensitivity screening and relative sensitivity calculation

All mice were tested for pain sensitivity using hot plate and von Frey filaments three times within one week before experimental injury to minimize habituation effects (pre-CCI), and then they were tested once seven days after surgery (post-CCI). To reduce inter-animal variability, relative pain sensitivity was calculated for each animal by calculating the ratio between the post-CCI and average pre-CCI values (i.e. post-CCI/pre-CCI) of the thermal and mechanical thresholds. Thus, a low ratio indicates a low pain threshold and vice versa.

### Histology

Seven days after CCI, pain threshold measurements were performed and mice were then sacrificed by isoflurane narcosis and subsequent cervical dislocation. Sciatic nerves were dissected and embedded in either paraffin or epoxy resin.

For paraffin embedding, nerve sections including CCI-crush sites were fixed with 4% paraformaldehyde (PFA) and subsequently dehydrated with increasing ethanol concentrations. Xylol was used as intermediate substance before immersing nerve sections in warmed-up paraffin. Sections of 6-µm thickness were cut proximal, central and distal to a CCI lesion with a microtome (Reichert-Jung). Primary antibodies used for staining paraffin sections were rabbit polyclonal anti-human/mouse/rat S100-A1 (Sigma #SAB4502708; 1:2000), mouse monoclonal anti-mouse/rat MHC II (Serotec #MCA2687GA; 1:100), rabbit polyclonal anti-Neurofilament L (α-NFL; Chemicon International, Temecula, CA, #AB9568*;* 1:4000) and mouse anti-CD4 (Abcam #ab51312; 1:2000). All antibodies were diluted in antibody diluent (DAKO #S3022). Nuclei were stained with DAPI (included in used mounting medium with 1.5 µg/ml DAPI from vector #H-1200) or hemalum. Visualization was performed either with the help of the fluorescent secondary antibodies Alexa Fluor 488-conjugated goat anti-rabbit IgG (Invitrogen #A11034; 1:200 in PBS) and Alexa Fluor 594-conjugated goat anti-mouse IgG (Invitrogen #A11032; 1:200 in PBS) or DAB-based staining as previously described^10^. Stained sections were photographed on an Axioplan 2 microscope (Zeiss) with an AxioCam HRC camera (Zeiss). Pictures of DAB-stained nerve sections were merged using Adobe Photoshop CS3 (Adobe Systems) and ImageJ was used to manually count the total number of cell infiltrates as well as the number of CD4 positive cells per area. The fluorescence signal based on α-NFL staining was quantified as arbitrary units (AU) using integrated density measurements in ImageJ.

Sciatic nerves were epoxy resin embedded as previously described^45^. Semi-thin sections (0.5 µm) of sciatic nerves were cut 2 mm proximal to the most proximal CCI lesion site using an Ultracut E microtome (Reichert-Jung). Sections were Toluidine-Blue stained with 1% Borax (disodium tetraborate solution), mounted using a RotiHisto Kit II (Roth) according to manufacturer’s instructions and photographed at 63x magnification with an AxioCam HRC camera (Zeiss) on an Axioplan 2 microscope (Zeiss). Overlapping pictures of the entire nerve section were merged using Adobe Photoshop. Axon circumference and myelin circumference were manually measured for >100 axons per nerve section using ImageJ. The *g* ratio was calculated by dividing axon circumference by myelin circumference. The higher the *g* ratio, the thinner the myelin sheath of the axon^45^.

### Statistics

Data are presented as mean ± s.e.m. Data were compared using Student’s *t*-test for unrelated samples unless indicated otherwise in the figure legend. *p*-value < 0.05 was considered significant. GraphPad Prism 5 was used for statistical analysis.

## Electronic supplementary material

Supplementary information
