# Supplementary material for: Schwann cells promote post-traumatic nerve inflammation and neuropathic pain through MHC class II
Source: Sci Rep. 2017 Oct 2;7:12518. doi: 10.1038/s41598-017-12744-2 (PMC5624882; doi:10.1038/s41598-017-12744-2)
Supplement: Supplementary file 1 — Supplementary information [file 41598_2017_12744_MOESM1_ESM.pdf]

Supplementary information

for:

Schwann cells promote post-traumatic nerve inflammation and neuropathic pain through MHC class II.

Maike Hartlehnert[1,§], Angelika Derksen[2,§], Tim Hagenacker[3], David Kindermann[3], Maria Schäfers[3], Mathias Pawlak[4], Bernd C. Kieseier[2], Gerd Meyer zu Horste[1, 2,*]

1) Department of Neurology, University Hospital Münster, Münster, Germany
2) Department of Neurology, Heinrich-Heine-University, Medical Faculty, Düsseldorf, Germany
3) Department of Neurology, University of Duisburg-Essen, Germany.
4) Evergrande Center for Immunologic Diseases, Harvard Medical School and Brigham and Women's Hospital, Boston, MA, USA

§ These authors contributed equally to this study.

**Correspondence to:*
Gerd Meyer zu Hörste
Department of Neurology
Westfälische-Wilhelms-University
48149 Münster
Germany
gerd.mzh@uni-muenster.de

Running title: MHC class II on Schwann cells promotes post-traumatic neuropathic pain.

Supplementary Figure Legends

Supplementary Figure 1: Additional Characterization of the $P0^{Cre}IAb^{fl/fl}$ mouse line.

Longitudinal paraffin sections (thickness 6 µm) of the non-injured sciatic nerve from wildtype $IAb^{fl/fl}$ mice (top panels) and $P0^{Cre}IAb^{fl/fl}$ mice (bottom panels) were stained against S100 (green signal), MHC class II (MHC-II) (red signal) using fluorescently labelled secondary antibodies and nuclei were stained with DAPI. Right panels depict higher magnifications of the areas indicated in the left panels.

Supplementary Figure 2: Chronic constriction injury (CCI) of sciatic nerve.

Longitudinal paraffin embedded sciatic nerve sections from wildtype mice without CCI (top panel) and seven days after CCI (bottom panel) were stained against neurofilament light chain protein (NFL; green signal) using fluorescently labelled secondary antibodies and nuclei were stained with DAPI.

Supplementary Figure 3: Temporal scheme of the experiments.

Hot plate and von Frey filament testing were performed for three consecutive days and on the third day chronic constriction injury (CCI) of the sciatic nerve was elicited. Testing was repeated on day 7 after CCI and the animals were sacrificed for histological analysis.

S1

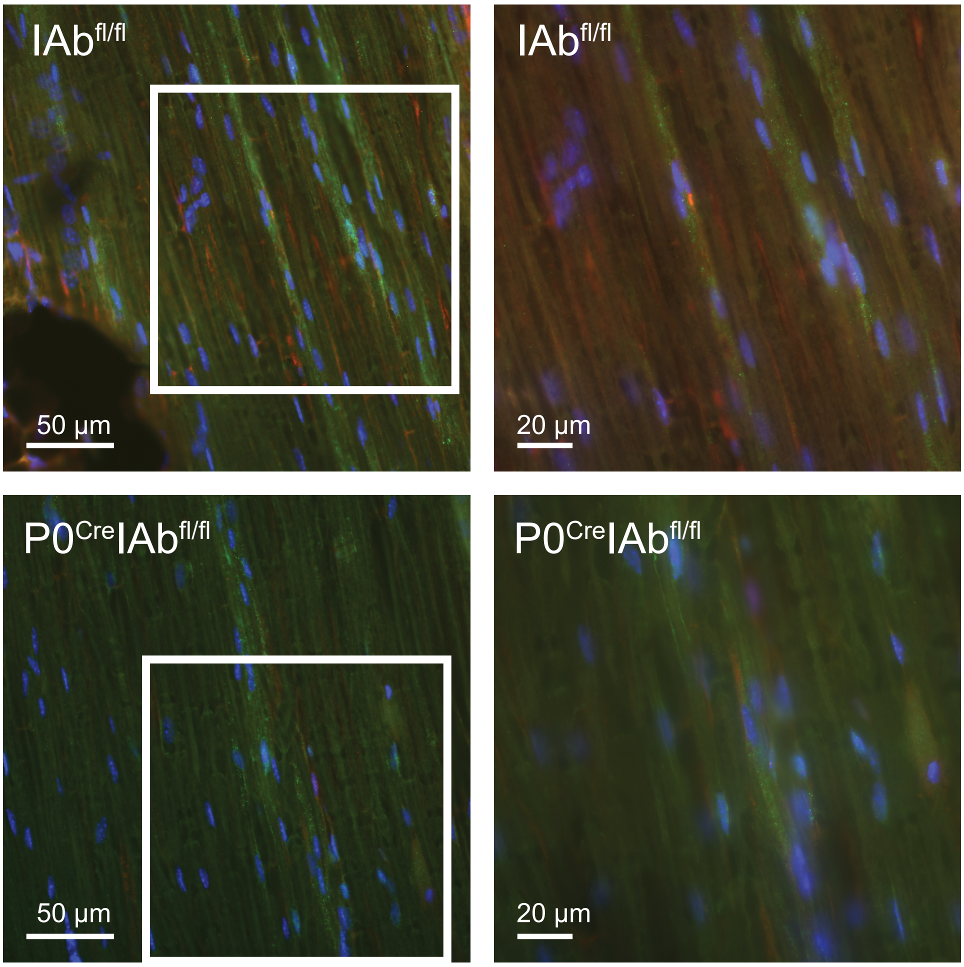
IAbfl/fl
50 µm
IAbfl/fl
20 µm
P0CreIAbfl/fl
50 µm
P0CreIAbfl/fl
20 µm

S2

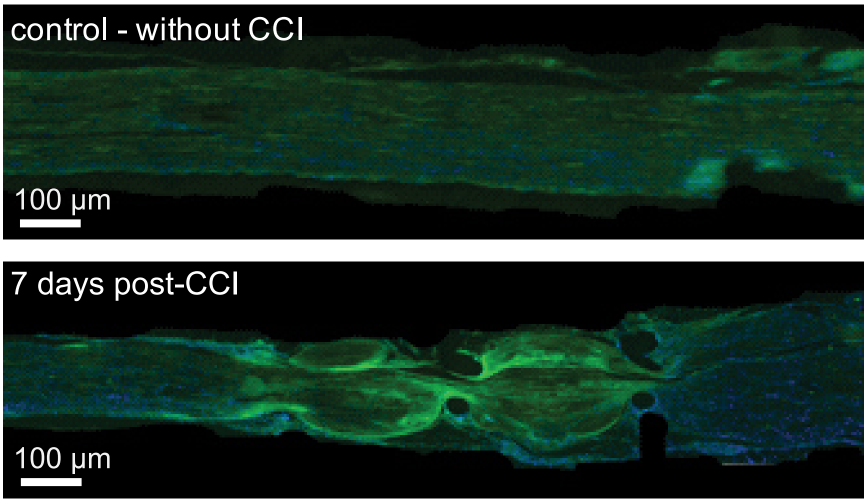
control - without CCI
100 µm
7 days post-CCI
100 µm

S3

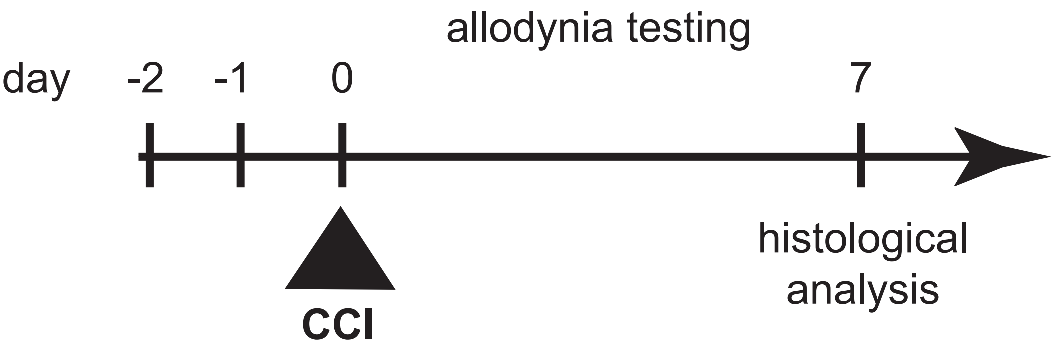
allodynia testing
day
-2
-1
0
7
histological analysis
CCI
